# Supplementary material for: A role for ascorbate conjugates of (+)-catechin in proanthocyanidin polymerization
Source: Nat Commun. 2022 Jun 14;13:3425. doi: 10.1038/s41467-022-31153-2 (PMC9197940; doi:10.1038/s41467-022-31153-2)
Supplement: Supplementary file 1 — Supplementary Information [file 41467_2022_31153_MOESM1_ESM.pdf]

A role for ascorbate conjugates of (+)-catechin in proanthocyanidin polymerization

Yu *et al.*

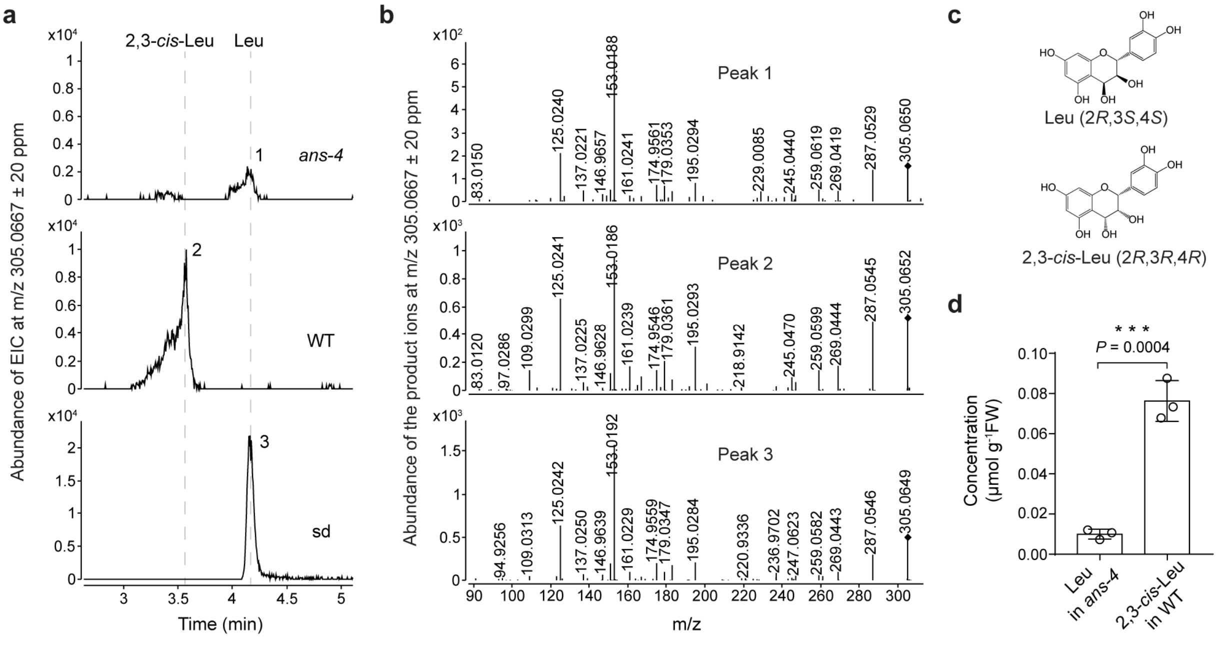

Supplementary Figure 1. Measurement of leucocyanidin isomers in 7 DAP siliques of the *A. thaliana ans-4* mutant and wild-type (WT).

a, Extracted ion chromatogram (EIC) of leucocyanidin isomers from soluble extracts of 7 DAP siliques of the *A. thaliana ans-4* mutant and WT plants on UHPLC-QToF.

b, Product ions of compounds marked in **a**.

c, Chemical structures of the two leucocyanidin isomers measured.

d, The concentration of leucocyanidin isomers in *ans-4* mutant and WT soluble extracts. FW: fresh weight. Data are shown as the mean ± SD (for n = 3 biologically independent samples; *** $P < 0.001$, two-tailed unpaired Student's *t* tests).

Leu, leucocyanidin (with 2,3-*trans* conformation); 2,3-*cis*-Leu, 2,3-*cis*-leucocyanidin; sd, standard. Leu is identified by reference to the retention time and MS/MS information of sd, and the annotation of 2,3-*cis*-Leu is based on the MS/MS information and the earlier retention time than Leu on reverse-phase UHPLC shown in our previous study [16]. Source data of Supplementary Figure 1d are provided as a Source Data file.

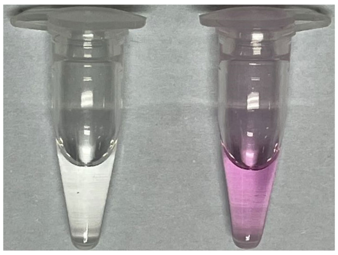

Without heat After heat

Supplementary Figure 2. Butanolysis of Cys-C.

One hundred µL of butanolysis buffer with 0.1 mM Cys-C standard was incubated in 50°C for 1 h and yielded red color product (right panel), while the system without heat treatment remained no colored (left panel).

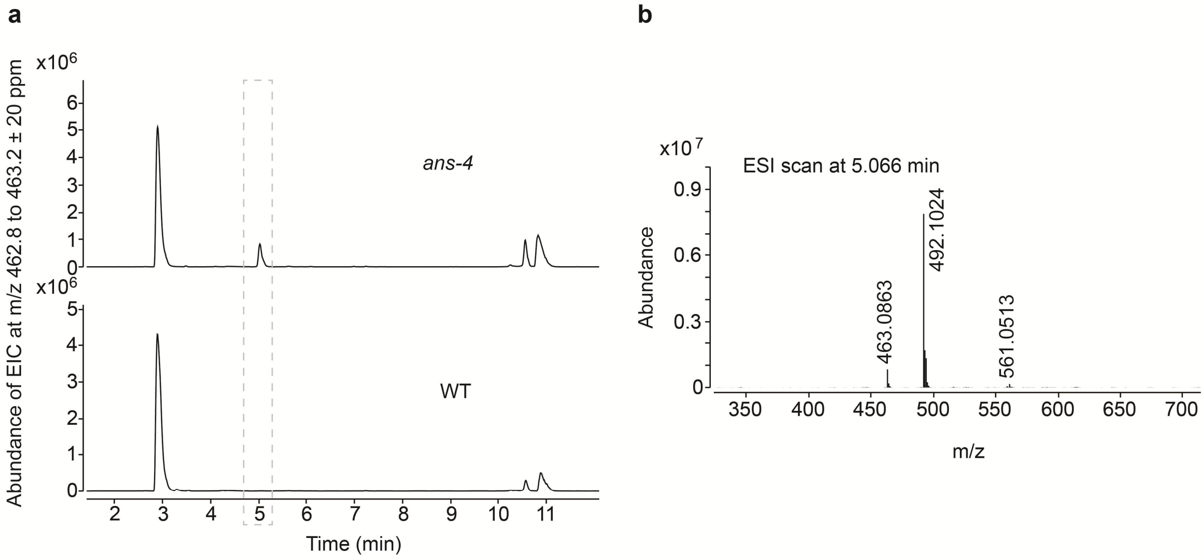

Supplementary Figure 3. Identification by high resolution UHPLC-QToF mass spectrometry of the putative catechin conjugate with m/z 463 in the low resolution mass spectrum.

a, Identification of the retention time for the candidate with m/z 463 in UHPLC-QToF.

b, Accurate mass analysis shows that the m/z of the candidate is 463.0863 ± 20 ppm.

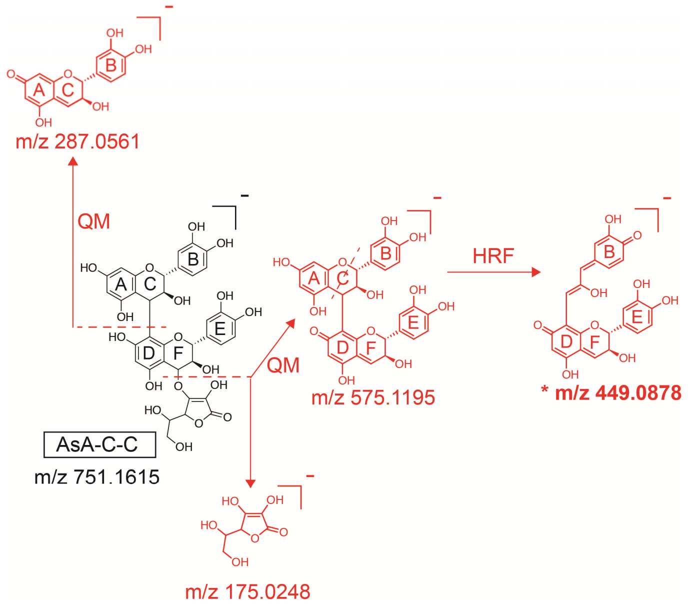

Supplementary Figure 4. Fragmentation mode deduction supporting the conclusion that compounds 1 and 3 in Fig. 5a and Fig. 5b of the main text are AsA-C-C.

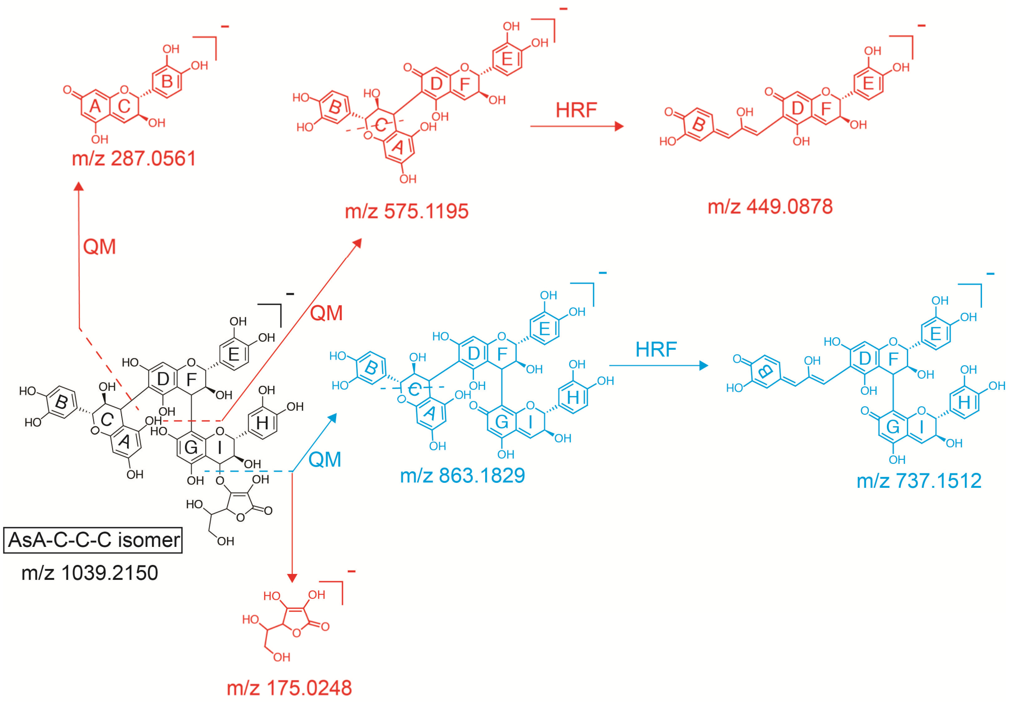

Supplementary Figure 5. Fragmentation mode deduction supporting the conclusion that compounds 6 and 7 in Fig. 5c and Fig. 5d of the main text are AsA-C-C-C isomers.

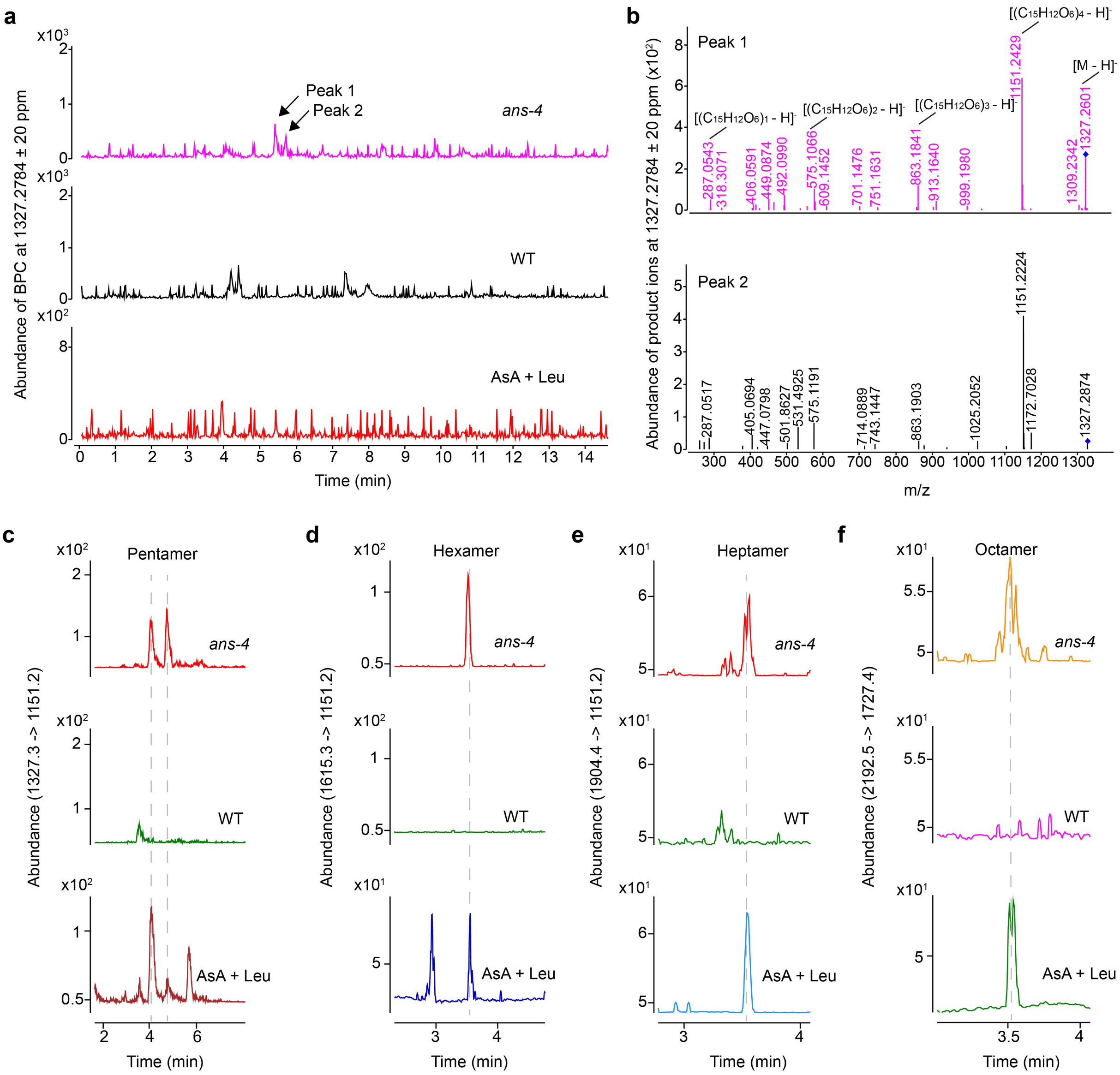

Supplementary Figure 6. AsA-[C]$_n$ pentamer to octamer accumulate in 7 DAP siliques of the *A. thaliana ans-4* mutant.

The soluble PA fraction of 7 DAP siliques of the *A. thaliana ans-4* mutant was analyzed in parallel with the product of reaction of AsA + leucocyanidin (Leu) as the standard using UHPLC-QToF or UHPLC-QqQ.

a, Base peak chromatogram (BPC) showing that two compounds with the same m/z (1327.2784 ± 20 ppm) as AsA-[C]$_n$ pentamer accumulate in the *A. thaliana ans-4* mutant.

b, Product ions of compounds marked in **a**. A series of fragments of $[(C_{15}H_{12}O_6)_m - H]^-$ ($0<m\leq n$) will result from QM fission during the IFL cleavage or the leaving of the AsA moiety.

c to **f**, Compound retention times on UHPLC and MRM transitions on QqQ suggesting that both *ans-4* and AsA+Leu reaction possess AsA-[C]$_n$ pentamer to octamer that do not exist in the WT.

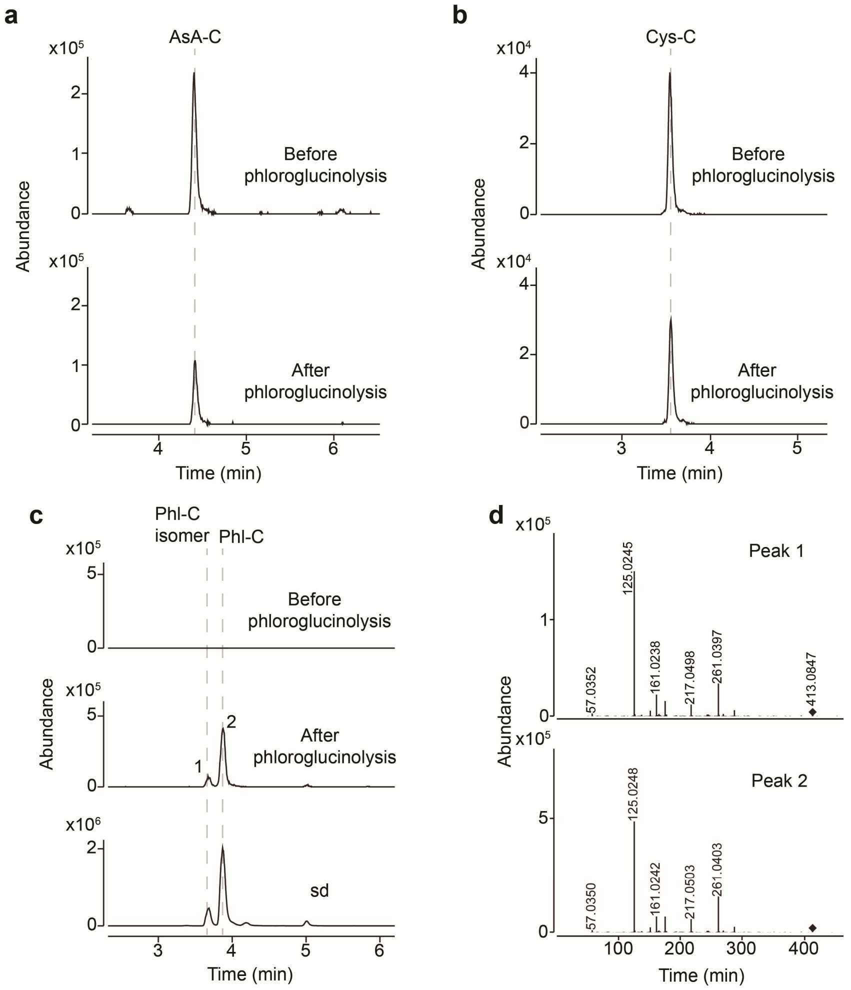

Supplementary Figure 7. AsA-C and Cys-C are partially cleaved under phloroglucinolysis.

Fifty µL of aqueous solution containing equimolar (10 µM) amounts of AsA-C and Cys-C was lyophilized and then re-dissolved in 100 µL phloroglucinolysis buffer. To prepare the sample for measuring target compounds before phloroglucinolysis, the lysis system was dried immediately by nitrogen flow and then re-dissolved in 100 µL 50% (v/v) methanol/water. To prepare the sample for measuring target compounds after phloroglucinolysis, the lysis system was incubated at 50°C for 30 min, followed by nitrogen flow drying and re-dissolving in 100 µL 50% (v/v) methanol/water.

a, UHPLC-QToF analysis showing that levels of AsA-C (EIC at m/z 463.0882 ± 20 ppm) were reduced after phloroglucinolysis.

b, UHPLC-QToF analysis showing that levels of Cys-C (EIC at m/z 408.0759 ± 20 ppm) were reduced after phloroglucinolysis.

c, UHPLC-QToF analysis (EIC at m/z 413.0878 ± 20 ppm) showing that phloroglucinolysis in the presence of AsA-C and Cys-C gave rise to phloroglucinol-catechin adduct (Phl-C) and phloroglucinol-catechin isomer (Phl-C isomer), which was supported by comparing the retention time of phloroglucinolysis products of procyanidin B3 as the standard (sd) and the MS2 profile in **d**.

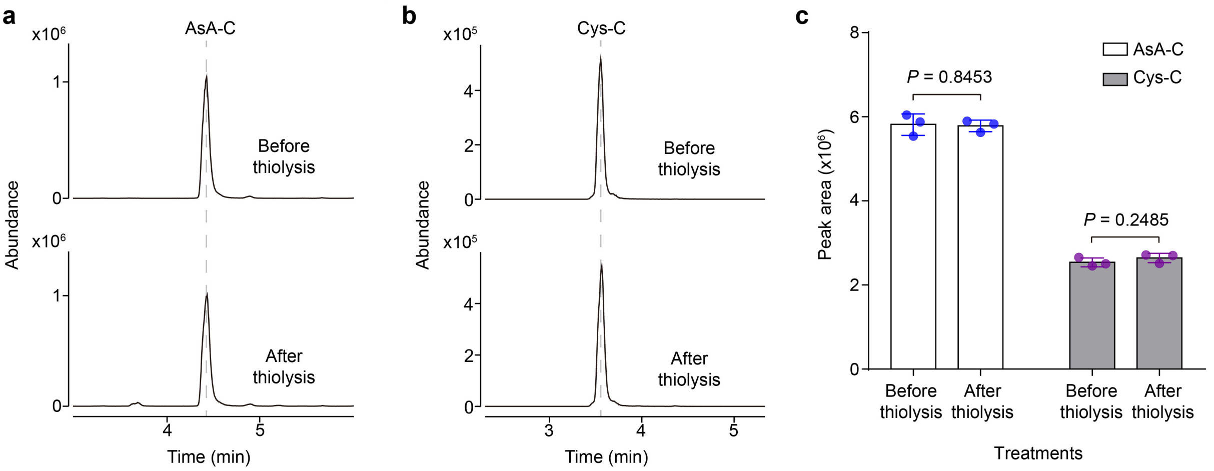

Supplementary Figure 8. AsA-C and Cys-C are stable under thiolysis in the presence of excess Cys. Fifty µL of aqueous solution containing equimolar (10 µM) amounts of AsA-C and Cys-C was lyophilized and then re-dissolved in 100 µL thiolysis buffer. To prepare the sample for measuring target compounds before thiolysis, the lysis system was dried immediately by nitrogen flow and then re-dissolved in 100 µL 50% (v/v) methanol/water. To prepare the sample for measuring target compounds after thiolysis, the lysis system was incubated at 50°C for 30 min, followed by nitrogen flow drying and re-dissolving in 100 µL 50% (v/v) methanol/water.

a, UHPLC-QToF analysis of AsA-C (EIC at m/z 463.0882 ± 20 ppm) before and after thiolysis.

b, UHPLC-QToF analysis of Cys-C (EIC at m/z 408.0759 ± 20 ppm) before and after thiolysis.

c, Quantification and statistical analysis of the abundance of AsA-C and Cys-C before and after thiolysis. Data are shown as the mean ± SD (for n = 3 independent replicates); the P values were calculated from two-tailed unpaired Student's t tests.

Source data of Supplementary Figure 8c are provided as a Source Data file.

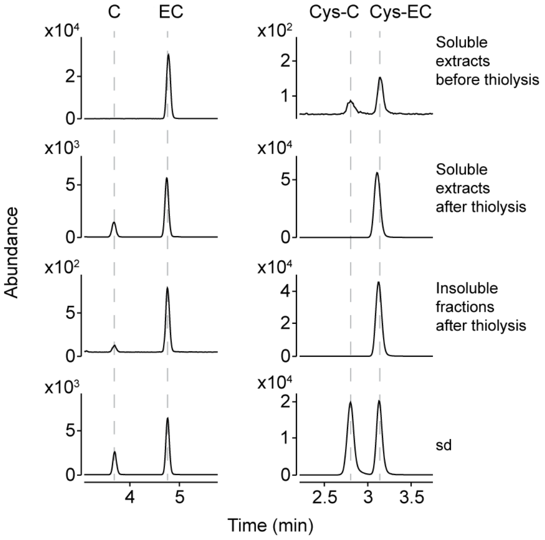

Supplementary Figure 9. Thiolysis analysis of the soluble extract and insoluble fraction of 7 DAP siliques of *A. thaliana* WT in the presence of excess Cys.

UHPLC-QqQ analysis of (+)-catechin (denoted as "C") and (-)-epicatechin (denoted as "EC") with MRM transition of m/z (289.1 → 123.1); UHPLC-QqQ analysis of Cys-C and Cys-EC with MRM transition of m/z (408.1 → 125.0). sd: standard. Note that the ion abundances are not directly comparable among different type of samples, as different dilution factors were applied to each sample to prevent the over-saturation of ion abundance in the QqQ detector.

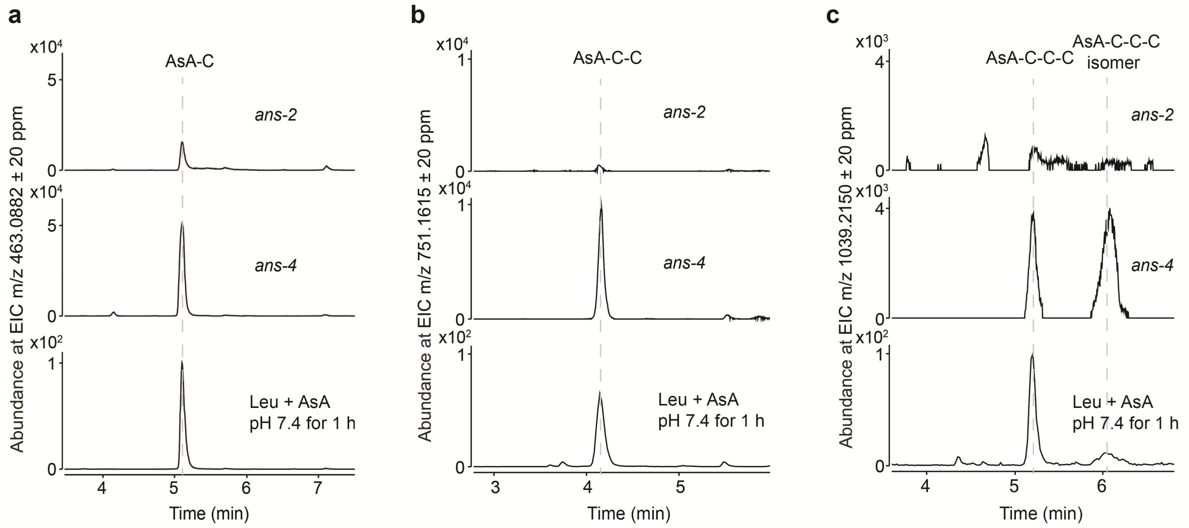

Supplementary Figure 10. **Targeted analysis of AsA-[C]$_n$ in 7 DAP siliques of independent weak (*ans-2*) and strong (*ans-4*) *A. thaliana ans* mutant alleles using UHPLC-QToF.**

a, Targeted analysis of AsA-C using EIC at m/z 463.0882 ± 20 ppm.

b, Targeted analysis of AsA-C-C using EIC at m/z 751.1615 ± 20 ppm.

c, Targeted analysis of AsA-C-C-C using EIC at m/z 1039.2150 ± 20 ppm.

The product of the *in vitro* reaction of AsA + leucocyanidin (Leu) was analyzed in parallel as the standard.

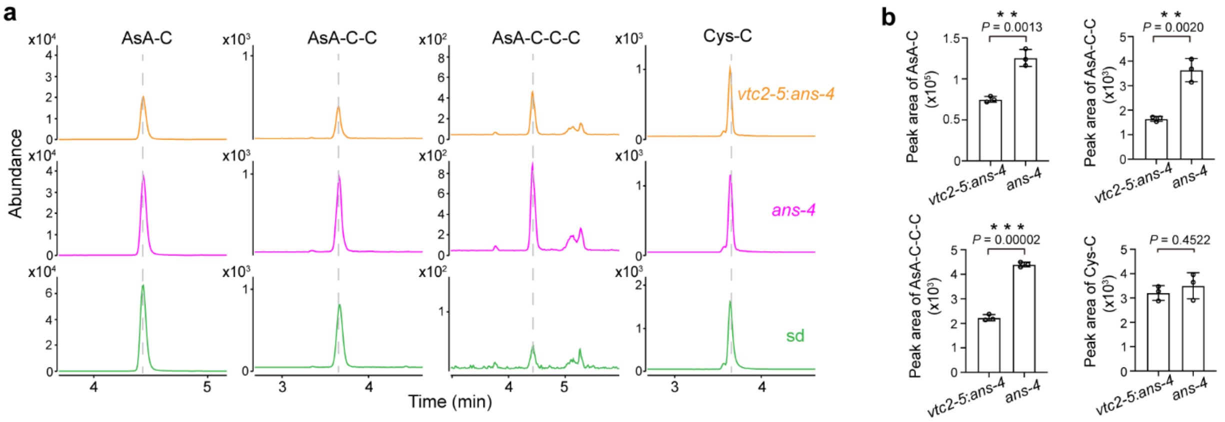

Supplementary Figure 11. Detection and quantification of AsA-[C]$_n$ and Cys-C in 7 DAP siliques of *A. thaliana vtc2-5*:*ans-4 and ans-4* mutants using UHPLC-QqQ.

a, Targeted analysis of AsA-[C]$_n$ and Cys-C in 7 DAP siliques of *A. thaliana vtc2-5*:*ans-4* and *ans-4* mutants. MRM transitions of m/z (463.1 → 175.0), m/z (751.0 → 287.0), m/z (1039.0 → 863.0) and m/z (408.1 → 125.0) were used for the detection of AsA-C, AsA-C-C, AsA-C-C-C and Cys-C respectively.

b, Quantification and statistical analysis of the abundance of AsA-[C]$_n$ and Cys-C in 7 DAP siliques of *A. thaliana vtc2-5*:*ans-4* and *ans-4* mutants. Data are shown as the mean ± SD (for n = 3 biologically independent samples); the *P* values were calculated from two-tailed unpaired Student's *t* tests. ** $P < 0.01$, *** $P < 0.001$.

sd: standard. Source data of Supplementary Figure 11b are provided as a Source Data file.

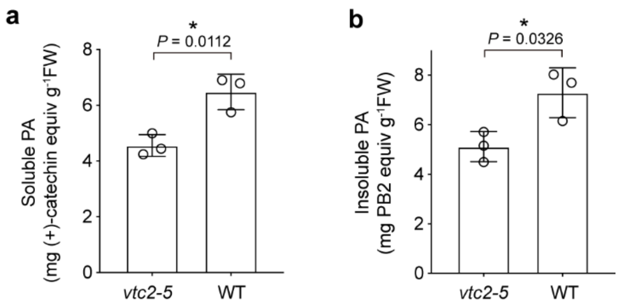

Supplementary Figure 12. **Analysis of PA content in 7 DAP siliques of *A. thaliana vtc2-5* mutant and WT.**

a, Soluble PA levels were measured by using dimethylaminocinnamaldehyde (DMACA) reagent and expressed as (+)-catechin equivalents.

b, Insoluble PA levels were determined by the butanol-HCl method and expressed as procyanidin B2 (PB2) equivalents.

In the bar plots, data are shown as the mean ± SD (for n = 3 biologically independent samples); the *P* values were calculated from two-tailed unpaired Student's *t* tests. * $P < 0.05$. FW, fresh weight. Source data of Supplementary Figure 12a and b are provided as a Source Data file.

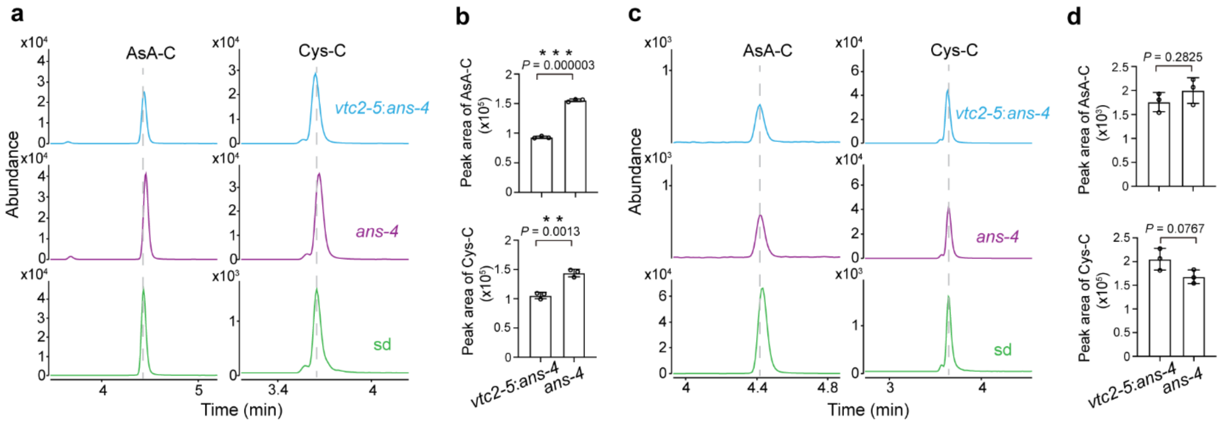

Supplementary Figure 13. Thiolysis analysis of soluble extracts and insoluble fraction of 7 DAP siliques of *A. thaliana vtc2-5*:*ans-4* and *ans-4* mutants in the presence of excess Cys.

a, UHPLC-QqQ analysis of AsA-C and Cys-C in the thiolysis products of soluble extracts using MRM transition of m/z (463.1 → 175.0) and m/z (408.1 → 125.0) respectively.

b, Quantification and statistical analysis of the abundance of AsA-C and Cys-C in the thiolysis products of soluble extracts from 7 DAP siliques of *A. thaliana vtc2-5*:*ans-4* and *ans-4* mutants.

c, UHPLC-QqQ analysis of AsA-C and Cys-C in the thiolysis products of insoluble fractions using MRM transition of m/z (463.1 → 175.0) and m/z (408.1 → 125.0) respectively.

d, Quantification and statistical analysis of the abundance of AsA-C and Cys-C in the thiolysis products of insoluble fractions from 7 DAP siliques of *A. thaliana vtc2-5*:*ans-4* and *ans-4* mutants.

Data are shown as the mean ± SD (for n = 3 biologically independent samples); the *P* values were calculated from two-tailed unpaired Student's *t* tests. ** $P < 0.01$, *** $P < 0.001$. sd: standard. Source data of Supplementary Figure 13b and d are provided as a Source Data file.

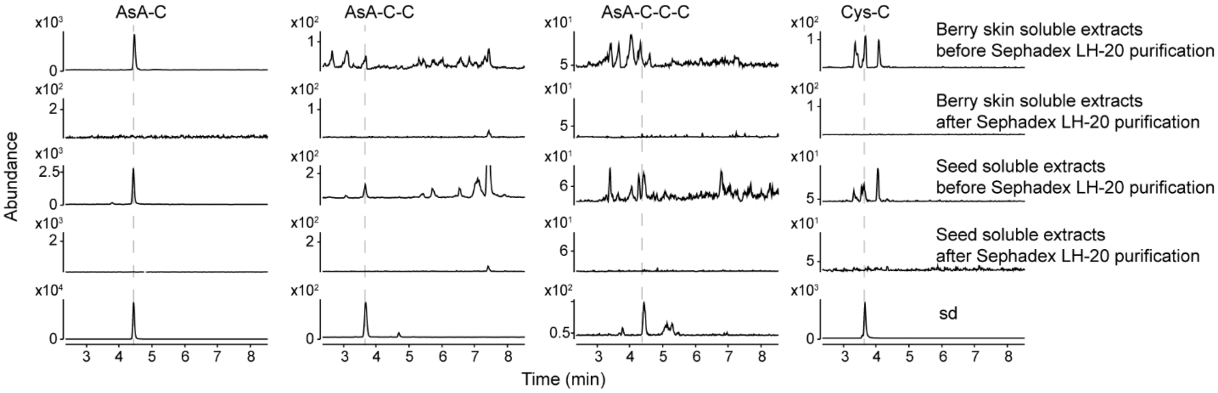

Supplementary Figure 14. UHPLC-QqQ analysis of AsA-[C]$_n$ and Cys-C in the soluble extracts from grape berries and seeds of E-L 31 stage before and after Sephadex LH-20 resin purification. MRM transitions of m/z (463.1 → 175.0), m/z (751.0 → 287.0), m/z (1039.0 → 863.0) and m/z (408.1 → 125.0) were used for the detection of AsA-C, AsA-C-C, AsA-C-C-C and Cys-C respectively. sd: standard.

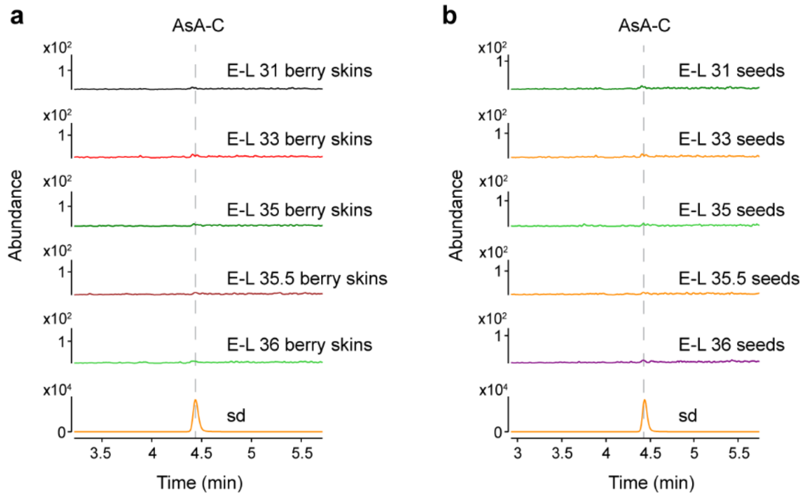

Supplementary Figure 15. UHPLC-QqQ analysis of AsA-C in the thiolysis products of the insoluble fraction of grape berry skins and seeds at different E-L developmental stages.

a, UHPLC-QqQ analysis of AsA-C with MRM transition of m/z (463.1 → 175.0) in the thiolysis products of the insoluble fractions of grape berry skins at the indicated developmental stages.

b, UHPLC-QqQ analysis of AsA-C with MRM transition of m/z (463.1 → 175.0) in the thiolysis products of the insoluble fractions of grape seeds at the indicated developmental stages.

sd: standard.

Supplementary Table 1. Predictions of the bond dissociation energies of the compounds related to (+)-catechin conjugates.

Compounds	Output Structure from ALFABET	Bond Type	BDE (kcal mol^{-1})
AsA-C-C-C	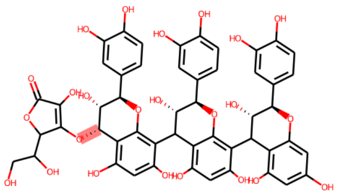	C-O	51.2
AsA-C-C	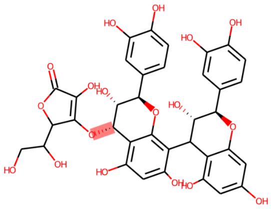	C-O	51.2
AsA-C	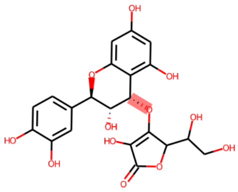	C-O	51.6
Cys-C	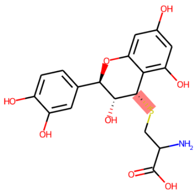	C-S	64.7
Methyl-*O*-Catechin	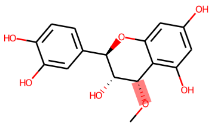	C-O	79.3
AsA	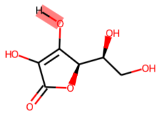	H-O	79.5
Cys	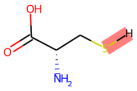	H-S	85.1
Methanol	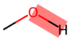	H-O	102.0

The dissociation energy of the bond (highlighted in red) in each compound is predicted using ALFABET BDE Estimator (https://bde.ml.nrel.gov/) [35]. BDE, bond dissociation energy.

Supplementary Table 2. Sequences of primers used in this work

Primer name	Sequence (5'-3')	Purpose
ans-2-LP	TTCCCCTGTTTTTAAGTTTATTT	Genotyping
ans-2-RP	AGAAAGACACAAACACATTATAAA	Genotyping
ans-4-LP	CTGCTTTGAAAGAAGGCACAC	Genotyping
ans-4-RP	AGCCGGAGAAGAGTTTTTCAG	Genotyping
vtc2-5-LP	GTGTTCTTGACTGCTTGCCTC	Genotyping
vtc2-5-RP	CCAAGAAGCTTCAAATGCAAC	Genotyping
LBb1.3	ATTTTGCCGATTTCGGAAC	Genotyping
