# Supplementary material for: A role for ascorbate conjugates of (+)-catechin in proanthocyanidin polymerization
Source: Nat Commun. 2022 Jun 14;13:3425. doi: 10.1038/s41467-022-31153-2 (PMC9197940; doi:10.1038/s41467-022-31153-2)
Supplement: Supplementary file 3 — Reporting Summary [file 41467_2022_31153_MOESM3_ESM.pdf]

nature portfolio

Corresponding author(s): Changqing Duan

Last updated by author(s): May 25, 2022

Reporting Summary

Nature Portfolio wishes to improve the reproducibility of the work that we publish. This form provides structure for consistency and transparency in reporting. For further information on Nature Portfolio policies, see our Editorial Policies and the Editorial Policy Checklist.

Statistics

For all statistical analyses, confirm that the following items are present in the figure legend, table legend, main text, or Methods section.

n/a	Confirmed	
☐	☒	The exact sample size (n) for each experimental group/condition, given as a discrete number and unit of measurement
☐	☒	A statement on whether measurements were taken from distinct samples or whether the same sample was measured repeatedly
☐	☒	The statistical test(s) used AND whether they are one- or two-sided<br>*Only common tests should be described solely by name; describe more complex techniques in the Methods section.*
☐	☒	A description of all covariates tested
☐	☒	A description of any assumptions or corrections, such as tests of normality and adjustment for multiple comparisons
☐	☒	A full description of the statistical parameters including central tendency (e.g. means) or other basic estimates (e.g. regression coefficient) AND variation (e.g. standard deviation) or associated estimates of uncertainty (e.g. confidence intervals)
☐	☒	For null hypothesis testing, the test statistic (e.g. F, t, r) with confidence intervals, effect sizes, degrees of freedom and P value noted<br>*Give P values as exact values whenever suitable.*
☒	☐	For Bayesian analysis, information on the choice of priors and Markov chain Monte Carlo settings
☒	☐	For hierarchical and complex designs, identification of the appropriate level for tests and full reporting of outcomes
☒	☐	Estimates of effect sizes (e.g. Cohen's d, Pearson's r), indicating how they were calculated

Our web collection on statistics for biologists contains articles on many of the points above.

Software and code

Policy information about availability of computer code

Data collection	LC/MS data was collected using Acquisition module of Agilent MassHunter Workstation B.10.0 and Microsoft Excel 2016.
Data analysis	LC/MS data was analyzed using Quantitative Analysis module of Agilent MassHunter Workstation B.10.0. Statistical tests were carried out with GraphPad Prism 7.0. Bond dissociation energies were predicted using ALFABET BDE Estimator (https://bde.ml.nrel.gov/).

For manuscripts utilizing custom algorithms or software that are central to the research but not yet described in published literature, software must be made available to editors and reviewers. We strongly encourage code deposition in a community repository (e.g. GitHub). See the Nature Portfolio guidelines for submitting code & software for further information.

Data

Policy information about availability of data

All manuscripts must include a data availability statement. This statement should provide the following information, where applicable:

- Accession codes, unique identifiers, or web links for publicly available datasets
- A description of any restrictions on data availability
- For clinical datasets or third party data, please ensure that the statement adheres to our policy

All data needed to evaluate the conclusions in the paper are present in the paper and the Supplementary Information. Raw LC/MS data have been deposited to the EMBL-EBI MetaboLights database (https://www.ebi.ac.uk/metabolights/) with the identifier MTBLS4942. Source Data are provided with this paper.

Field-specific reporting

Please select the one below that is the best fit for your research. If you are not sure, read the appropriate sections before making your selection.

☒ Life sciences ☐ Behavioural & social sciences ☐ Ecological, evolutionary & environmental sciences

For a reference copy of the document with all sections, see nature.com/documents/nr-reporting-summary-flat.pdf

Life sciences study design

All studies must disclose on these points even when the disclosure is negative.

Sample size	Sample size was determined following the previous publication similar to this study. For each measurement of PAs content or composition in Arabidopsis, at least 100 developing siliques were collected and pooled from at least 30 independent plants. For each measurement of PAs composition in grape berry tissues, at least 300 flowers or berries were sampled and pooled from 70 different vines.
Data exclusions	There were no data exclusions.
Replication	The samples (plants) were collected from different batches and analyzed (with or without technical replicates for each). We have utilized the methods previously reported and frequently used. All attempts at replication were successful.
Randomization	Arabidopsis plants were grouped by genotype and were grown at the same time in pots in the same trays in the growth chambers. Grape samples were collected from the commercial vineyard and grouped by development stages. The clusters were randomly selected from 35 different vines located on the vineyard's north site and 35 different vines on the south site. Grape flowers and berries were randomly collected from the sunny and shady sides of the clusters.
Blinding	In addition to using different batch of samples as described above, Arabidopsis plants were selected randomly without consideration of genotype until the point of data analysis, and the grape sampling was conducted by the specialist on viticulture of grapevine unfamiliar with the project design.

Reporting for specific materials, systems and methods

We require information from authors about some types of materials, experimental systems and methods used in many studies. Here, indicate whether each material, system or method listed is relevant to your study. If you are not sure if a list item applies to your research, read the appropriate section before selecting a response.

Materials & experimental systems

n/a	Involved in the study	
☒	☐	Antibodies
☒	☐	Eukaryotic cell lines
☒	☐	Palaeontology and archaeology
☒	☐	Animals and other organisms
☒	☐	Human research participants
☒	☐	Clinical data
☒	☐	Dual use research of concern

Methods

n/a	Involved in the study	
☒	☐	ChIP-seq
☒	☐	Flow cytometry
☒	☐	MRI-based neuroimaging
